# Intervention Based Exclusively on Stage-Matched Printed Educational Materials Regarding Healthy Eating Does Not Result in Changes to Adolescents' Dietary Behavior

**DOI:** 10.1100/2012/174640

**Published:** 2012-04-01

**Authors:** Natacha Toral, Betzabeth Slater

**Affiliations:** ^1^Nutrition Department, Health Sciences School, University of Brasília, Campus Universitário Darcy Ribeiro, Asa Norte, 70910-900 Brasília, DF, Brazil; ^2^Nutrition Department, Public Health School, University of Sao Paulo, Avenida Dr. Arnaldo, 715, 2 No. andar, 01246-904 São Paulo, SP, Brazil

## Abstract

*Purpose.* To assess the impact of a six-month stage-based intervention on fruit and vegetable intake, regarding perceived benefits and barriers, and self-efficacy among adolescents. *Design.* Randomized treatment-control, pre-post design. *Subjects/Setting.* Schools were randomized between control and experimental groups. 860 adolescents from ten public schools in Brasília, Federal District, Brazil were evaluated at baseline; 771 (81%) completed the study. *Intervention.* Experimental group received monthly magazines and newsletters aimed at promotion of healthy eating. *Measures.* Self-reported fruit and vegetable intake, stages of change, self-efficacy and decisional balance scores were evaluated at baseline and post-intervention in both groups. *Analysis.* The effectiveness of the intervention was evaluated using the analysis of covariance model (ANCOVA) and repeated measurement analysis by means of weighted least squares. Comparison between the proportions of adolescents who advanced through the stages during the intervention was performed using the Mantel-Haenszel chi-square test. *Results.* After adjusting for sex and age, study variables showed no modifications through the proposed intervention. There was no statistical difference in participant mobility in the intervention and control groups between the stages of change, throughout the study. *Conclusion.* A nutritional intervention based exclusively on distribution of stage-matched printed educational materials was insufficient to change adolescents' dietary behavior.

## 1. Introduction

The most common description of the dietary practices currently adopted during adolescence consists of fat, sugar, and sodium-rich diets, with little addition of fruits and vegetables [1, 2]. This picture correlates with increasingly early appearance of chronic diseases among adolescents, such as obesity and diabetes, which have a great public health impact [3]. According to the National Survey on Students' Health, conducted in Brazil in 2009, about one third of adolescents reported eating fruits and vegetables on at least five days of the week [4]. This intake level is much lower than the consumption of about 400 g of these foods a day that is recommended in order to prevent chronic diseases [3]. Therefore, there is an urgent need to implement strategies that can promote healthy dietary habits among adolescents.

In this regard, one possibility would be to adopt interventions based on behavioral theories, such as the transtheoretical model. This model makes it possible to determine *stages of change*, which represent *when *the behavioral change occurs and what the individual's degree of motivation toward accomplishing it is. In applying the theory to dietary behavior, individuals can be classified in one of five stages of change [5], namely: *precontemplation,* when they do not intend to change their dietary habits in the near future; *contemplation*, when they acknowledge that their dietary habits are inadequate, but still see difficulty in changing their habits; *preparation, *when they have decided to change their dietary habits within the next thirty days; *action, *when they have already changed some dietary habits over the last few months; *maintenance, *when they have kept to the changes for over six months.

In addition to the stages of change, the model encompasses three other constructs: the *decisional balance*, which deals with the balance that individuals make between the benefits and barriers relating to having a healthy diet; *self-efficacy*, which refers to individuals' self-confidence regarding their real ability to change their dietary behavior; the *processes of change*, which are individuals' experiences and feelings relating to advancing through the stages of change [5].

Since the basis for understanding the processes of change at different times was established, several studies among young people have shown positive results from nutritional interventions directed toward the stages of change. In addition to advancing through the stages with tailored interventions, individuals perceive more benefits and fewer barriers, present greater self-efficacy, and start to have a healthier diet, including greater fruit and vegetable intake [6–9].

## 2. Purpose

The objective of this study was to assess the impact of a stage-based intervention using printed educational materials, directed toward greater fruit and vegetable intake, along with the decisional balance relating to the benefits and barriers perceived and the self-efficacy, among adolescents in public schools in Brasília, Federal District, Brazil.

## 3. Methods

### 3.1. Design

This was a randomized trial in which a six-month nutritional intervention was developed during the 2009 school year. Two groups were assessed (intervention and control) in order to identify the effects of an intervention based on printed educational materials for promoting healthy dietary habits, which were distributed to the adolescents every month, both in classrooms and by mail. The materials were directed toward the participants' stages of change. This study was approved by the Research Ethics Committee of the School of Public Health, University of São Paulo.

### 3.2. Sample

The sampling process aimed toward obtaining a representative sample of adolescents in the 7th and 8th school years in Brasília. In order to calculate this, the 2006 School Census was used: at that time, there were 15 public schools in Brasília with 7th and 8th year students, with a total of 5,785 students enrolled [10]. In addition, the following statistical factors were used: a significance level of 5%, test power of 80%, correlation coefficient of 0.55 between observations at the two times and effect size of 0.10 between the null hypothesis of equal means in the two groups, and the alternative hypothesis of different means in the two groups. Thus, the minimum number of participants was estimated to be 706, with 353 in each group (control and intervention). Schools (not students) were randomized to avoid the possibility of exchanging experiences within the same social group. Four schools were randomly selected for the intervention group and six schools for the control group in order to achieve the minimum number calculated. All 7th and 8th year students in the ten schools were invited to participate in the study.

### 3.3. Measures

In addition to age (in years), the variables of sex, nutritional status, and family income (as multiples of the Brazilian minimum monthly salary) were evaluated. Nutritional status was assessed from weight and height measurements, which were taken in duplicate to investigate the body mass index according to age [11].

#### 3.3.1. Self-Reported Fruit and Vegetables Consumption

Initially, each adolescent was introduced to the concept of servings of fruits and vegetables by means of an illustrated leaflet. This showed that one serving of vegetables was equivalent to a dessert plate (or cup) of raw vegetables or half a dessert plate (or half a cup) of cooked vegetables, excluding potatoes and other starchy vegetables. In the case of fruits, for instance, one serving was equivalent to a medium-sized banana or orange, a small-sized apple, a medium-sized slice of pineapple or watermelon, a small-sized slice of papaya, or a small bunch of grapes. Next, the participants were asked about their habitual intake of such foods, in servings. Their answers were recorded in categories ranging from less than one serving a day to five or more servings a day.

In order to assess the adequacy of the fruit and vegetable intake, the recommended daily intake of these foods (400 g) [3], corresponding to about five servings a day, was taken into consideration. Thus, only the last option of self-reported consumption (five or more servings a day) was considered to be a healthy practice.

#### 3.3.2. Stages of Change

In order to classify the adolescents in the five stages of change regarding their fruit and vegetable consumption, an algorithm was used. This consisted of a questionnaire with a limited number of questions for which the answers are mutually exclusive, with a format that is similar to what has been described by several authors [8, 9, 12].

If the self-reported habitual fruit and vegetable consumption was inadequate, that is, less than five servings a day, the adolescents were asked about their intentions with regard to increasing their consumption of these foods in the near future. If they said that they had no intention of changing their dietary habits, they were classified as at the *precontemplation* stage. If they said that they did have some intention, they were classified as at the *contemplation* stage if they intended to increase their consumption of fruits and vegetables within the next six months or at the *preparation* stage if they showed an intention to increase their consumption within the next 30 days.

If the participants' habitual consumption of fruits and vegetables was adequate, that is, five or more servings a day, they were asked how long they had had this habit. If this was a recent habit, of less than six months, they were classified in the *action* stage; if they reported having this habit for six months or more, they were classified in the *maintenance *stage.

#### 3.3.3. Decisional Balance

A seven-item scale relating to the barriers faced by individuals when trying to eat in a healthy manner was included, along with a seven-item scale relating to the benefits identified by these individuals when following an adequate diet. Each item consisted of a sentence in which the participants expressed their agreement or disagreement with its contents, by means of a five-point Likert scale, ranging from 1—“I do not agree at all” to 5—“I strongly agree.” The items were selected from previous studies, which had shown relatively moderate-to-high internal consistency coefficients (Cronbach's *α*) [12–15], as well as a qualitative study on Brazilian adolescents who had spoken of their main difficulties in eating in a healthy manner [16].

#### 3.3.4. Self-Efficacy

This variable was assessed using a set of eight items. The participants expressed their agreement or disagreement with the content of these items on five-point Likert scales, ranging from 1—“I'm sure I cannot do this” to 5—“I'm sure I can do it.” The items were selected using the same criteria as presented regarding preparation of the scale for decisional balance [12, 14, 15].

#### 3.3.5. Previous Nutritional Guidance

In the initial assessment, all participants in the intervention group were asked whether they had received any nutritional guidance over the past year from a nutritionist or other healthcare professional.

### 3.4. Intervention

All adolescents in both the control and the intervention group were evaluated according to the variables presented above. After the six-month intervention, all the participants were assessed again. At the end of the first and second interviews, the participants in both groups received a folder presenting the 10 steps toward healthy eating during adolescence, as described by the Brazilian Ministry of Health [17], along with a record of their anthropometric measurements and their nutritional status at that time.

Every month, the intervention group received colorful printed magazines that promoted healthy eating and information newsletters directed toward the participants' stages of change that had been identified at the beginning of the study, with the aim of promoting fruit and vegetable consumption. The magazines were handed out at the schools and the newsletters were mailed.

The topics presented in the six magazines developed for the study were the means for overcoming the difficulties in eating healthily; the importance of breakfast and the right distribution of meals during the day; the relationship between nutritional status and adolescents' physical activity; the role of nutrients in adolescents' diets, the concepts of the terms *diet* and* light* and the importance of nutrition labeling; diet-related health problems such as diabetes, obesity, and eating disorders.

The basis for developing the magazines was the discourse of participants in a qualitative study conducted among focus groups that preceded this study [16]. The participants' testimonies were analyzed with a view to characterizing the educational materials for promoting healthy eating, particularly regarding their visual presentation and content, so that the material would be well accepted and understood by the adolescents.

Since previous studies found that high percentages of the adolescents were classified in the stages of change prior to action, that is, precontemplation and contemplation [2, 18], the magazines focused on the early stages. In this sense, the main objectives of the magazines were to increase knowledge about healthy eating through playful means, such as comics and games; to present ways to overcome barriers that hinder adoption of an appropriate diet and emphasize the benefits from following it, taking into account young people's lifestyles; to present curiosities about foods and healthy and practical recipes, along with tips on different cultural aspects of food, in such a way as to raise awareness about and reflect on diet and to increase interest in the subject.

The basis for developing the newsletters was stage-based materials for promoting regular consumption of fruits and vegetables that have been used in several studies, with satisfactory results among young adults [8, 9, 19, 20]. The newsletters took into account the *processes of change *needed to determine the progress from one stage to another [5].

The newsletters for the adolescents at the precontemplation stage covered the processes of change relating to *raising awareness, dramatic relief*, and *reevaluation of the environment*, so as to raise their awareness about the inadequacy of their intake of certain foods and promote acceptance that a dietary change was needed [21].

The materials for the adolescents classified in the contemplation and preparation stages included activities relating to the processes of change consisting of *self-reevaluation* and* self-liberation,* with strategies to increase their confidence in their own ability to improve their dietary habits, resolve their ambivalence about the commitment to change, and facilitate development of a specific plan for modifying their dietary behavior [21].

In the newsletters targeted toward individuals at the action and maintenance stages, the strategies emphasized consisted of maintaining an adequate intake of fruits and vegetables and preventing relapse (process of change: *stimulus control*), along with offering reward and reinforcement for keeping to the appropriate behavior (process of change: *contingency management*).

### 3.5. Analysis

Comparison of the variables between the control and intervention groups at the baseline was performed using the chisquare test for categorical data and Student's *t* test for independent samples for continuous variables.

The effect of the intervention was assessed by analyzing the changes in the components of the transtheoretical model (stages of change, decisional balance, and self-efficacy) and the changes in fruit and vegetable intake. Analysis of covariance (ANCOVA) models with adjustments for sex, age, and baseline values were used for each continuous outcome, that is, decisional balance between benefits and barriers and self-efficacy. A model for repeated measurement analysis with weighted minimum squares was used for categorized outcomes, that is, stages of change and fruit and vegetable intake. The same analyses were also performed with selection of only the group of adolescents that had not received nutritional guidance prior to the intervention.

The proportions of the adolescents who progressed through each stage of change over the course of the intervention were also assessed by means of Mantel-Haenszel chisquare analyses.

A 5% significance level was used. The analyses were conducted using the Statistical Analysis Software (SAS version 9.2; SAS Institute Inc., Cary, NC, USA) and the Statistical Package for the Social Sciences (SPSS for Windows, version 13.0; SPSS Inc., Chicago, Ill, USA).

The adolescents who participated in the survey had handed over a free and informed consent statement signed by their parents or guardians. This study was approved by the Research Ethics Committee of the School of Public Health, University of São Paulo.

## 4. Results

This study included initial participation by 860 students: 373 in the control group and 487 in the intervention group. In total, 771 participants completed the study, representing 81% followup. The losses were due to transfers of students to other schools (*n* = 54) and absence of participants on the days when interviews were conducted (*n* = 35).

More than half of the participants were aged between 11 and 13 years, and 59.5% were female (Table 1). It was found that over a third of the adolescents came from low-income families (less than two Brazilian minimum monthly salaries, that is, around US$ 460.00). It was observed that 18.1% were overweight to some extent and that only 10.9% reported consuming an adequate amount of fruits and vegetables (minimum of 5 servings a day).

At the baseline, there were no significant differences between the control and intervention groups regarding the category variables, with the exception of gender distribution, given that there was a higher percentage of girls in the control group (Table 1). There were also no significant differences between the groups regarding the decisional balance scores for barriers (*P* = 0.883) and benefits (*P* = 0.136), or regarding the self-efficacy scores (*P* = 0.196). Among the participants in the intervention group, it was found that 84.0% (*n* = 409) had not received any nutritional guidance before the start of the intervention.

The model for repeated measurements analysis using weighted minimum squares for categorized outcomes showed that the proposed intervention did not produce any significant changes to fruit and vegetable intake (*P* = 0.626) or to the stages of change (*P* = 0.905) (Table 2). Similarly, following the covariance analysis model applied to continuous variables, it was observed that the intervention did not result in any significant effects on the different variables of the transtheoretical model (Table 3). After adjusting for sex and age, no differences were observed between the control and intervention groups concerning the decisional balance for benefits (*P* = 0.288) or barriers (*P* = 0.415), or regarding participants' self-efficacy (*P* = 0.775).

The same analyses for assessing the impact of the intervention that were conducted on the full sample were also conducted by selecting only the participants who had not received prior nutritional guidance. This analysis showed that the absence of significant effects was maintained.

Comparing the control and intervention groups, there were no differences between the proportions of adolescents who progressed from precontemplation (*P* = 0.5613), contemplation (*P* = 0.2844) and preparation (*P* = 0.5779) to other stages, or between the proportions of the adolescents who remained in the action and maintenance stages (*P* = 0.4057). Thus, there were no statistical differences between the changes in either group regarding participants' mobility among the stages of change resulting from the proposed intervention.

## 5. Discussion

In the present study, high prevalence of inadequate fruit and vegetable intake among school children was observed, as also shown in several national studies conducted among Brazilian adolescents from different regions. [18, 22, 23] Likewise, studies conducted in many countries with different socioeconomic and cultural contexts, such as Sweden [1], Costa Rica [24], Australia [25], and USA [26], have also shown that dietary habits at this stage of life are frequently imbalanced.

This, together with the high percentage of overweight adolescents found in the present study, highlights the urgent need for impactful nutritional interventions in order to promote healthy dietary practices and motivate young people to adopt the nutritional recommendations. It should be emphasized that adolescence is an important period for establishing the dietary behavior that will be maintained in adulthood, and it is a stage in which there is greater independence in relation to choosing food, compared with childhood [27]. Overall, assessments of adolescent health have shown alarming results. Several Brazilian studies conducted among schoolchildren have shown high prevalence of cardiovascular risk factors at this early stage of life, reinforcing the importance of actions to promote health within the school environment [28–30].

It should be noted that the low fruit and vegetable intake among the participants in the present study may have been associated not only with the individuals' ages, but also with their families' socioeconomic conditions. Family income and schooling level have been seen to be determinants of the low intake of these foods in Brazil [31, 32]. According to Claro et al. [33], reducing the price of fruits and vegetables, which could be made possible through public policies and increases in family income, is one way to increase the participation of these foods in the Brazilian diet.

Unlike what was expected, the intervention proposed was ineffective in changing these adolescents' dietary behavior regarding their consumption of fruits and vegetables. Other studies with similar methodology have been conducted among young people, aimed at promoting the consumption of these foods, and have shown positive results after nutritional interventions targeted at stages of change. However, it should be noted that the major difference between the present study and other studies in the literature was that they included other nutritional intervention strategies in addition to distribution of educational materials to participants in order to promote fruit and vegetable consumption and change their dietary behavior [6, 8, 9].

According to Brinley et al. [6], an intervention with educational materials, lectures, and food tasting for six weeks resulted in advances through the stages of change for about 27% of the schoolchildren. In the study conducted by Richards et al. [8], the intervention among young students included sending personal newsletters directed toward the stages of change, along with motivational interviews and emails, over a four-month period. These authors found that the fruit and vegetable intake was significantly higher among the participants in the intervention group at the end of the study. Another nutritional intervention that lasted for six months included mailed printed materials targeted at the stages of change and two educational telephone calls to motivate change [9]. As a result, greater fruit and vegetable intake and more notable progression to the action and maintenance stages were observed in the intervention group.

As mentioned earlier, the printed materials used in studies by Richards et al. [8] and Nitzke et al. [9] were adopted as the basis for developing the materials of the present study. The original documents were accessed in full, prior to conducting the present study, with permission from the authors. The present study maintained a similar approach in the strategies used in the materials to promote consumption of fruits and vegetables, with adaptation of the language, content, and visual presentation for the Brazilian adolescent audience, given that the previous studies were directed towards young Americans aged 18 to 24 years.

It can, therefore, be said that additional motivation strategies should be included in parallel with intervention studies based on the distribution of printed materials tailored to the stages of change. Such strategies appear to be of fundamental importance for achieving a positive impact. On the other hand, recent criticism of the use of the transtheoretical model has suggested that interventions based on this theory are, in general, more intense and personalized than are the conditions presented to the control group. In other words, such interventions promote greater proximity and involvement between the researchers and the participants in the intervention group and contacts of greater frequency and of a more personal nature than in the control group. Consequently, the positive impact observed in intervention studies in this format could be a result from their intensity and not from the fact that they were based on the transtheoretical model [34].

Other important matters may explain the lack of impact from the proposed intervention in this study, such as the lack of family and teacher involvement in motivating behavioral changes. The family represents a fundamental social context in which behavior, actions, and habits of life are affected [35], including dietary practices. Families provide an important support network in the process of nutritional education for young people, especially in programs for prevention and treatment of obesity [36].

In addition, family participation in nutritional interventions is relevant given that often it is not the adolescent who makes decisions about the food available at home. The lack of autonomy in selecting foods that are consumed at home was shown in the study by Silva et al. [23], in which the mothers were responsible for the preparation of 44.7% of the food eaten by the students while the students were only responsible for 12.6% of the preparations.

Direct involvement by teachers in interventions is a debatable point, considering that Sichieri and Souza [37] showed that obesity prevention programs among children and adolescents are most effective when conducted by people devoted exclusively to this purpose than when conducted by teachers. This is because teachers are often involved in other school activities or are poorly motivated and do not receive specialized training and proper supervision for administering the intervention as proposed.

Likewise, the present study may not have had sufficient duration to promote changes in dietary behavior. The six-month duration was determined based on two points: the possibility of completing the study during the school year, from its submission to and approval by the school directors, until the final assessment of the students in both groups, and the fact that changing the stage from *action* to *maintenance* takes six months, which would make it possible to see any advance between the final stages.

Although the duration of previous interventions was less than or equal to six months, the literature has shown that studies of longer duration allow greater exposure of individuals to the information transmitted and favor behavioral changes [37]. However, it should be noted that long-term interventions imply higher costs and, when conducted in school environments, they compete with activities conducted in the classroom.

Another limitation of the present study relates to the assessment of food intake measurements through the categorized options. Small increases in the weekly consumption of fruits and vegetables that remained less than one serving (e.g., from two to four times a week) could not be identified through the evaluation adopted, since the first option corresponded to consumption of these foods “less than once a day.” Other forms of assessment of fruit and vegetable intake with greater sensitivity to small changes need to be incorporated, especially among groups in which there are significant percentages of individuals with inadequate dietary practices.

## 6. Conclusion

This study showed that a nutritional intervention exclusively based on distribution of printed educational materials targeted at individuals' stages of change was not enough to change the dietary habits of adolescents. No significant changes to the participants' fruit and vegetable intake, benefits, and barriers or perceived self-efficacy were identified. This indicates the complexity of the issues involved in adolescents' dietary behavior and emphasizes the major difficulties that need to be overcome in promoting healthy dietary practices in this group.

Interventions aimed at adolescents should be adapted so that, in addition to incorporating stage-matched printed educational materials, they also include other strategies for motivating them to adopt a healthy diet. It is also important to involve the social network that surrounds adolescents, in order to provide support for behavioral changes, and consider the duration of the nutritional intervention and the assessment measurements that can identify small changes in dietary habits.

## Figures and Tables

**Table 1:** Comparability between the intervention and control groups, regarding baseline characteristics.

Variables	Total		Control Group		Intervention Group		*P *value
	*n*	%	*n*	%	*n*	%	
Age							
11–13 yrs	489	56.9%	207	55.5%	282	57.9%	0.459
14–16 yrs	365	42.4%	163	43.7%	202	41.5%	
17–19 yrs	6	0.7%	3	0.8%	3	0.6%	

Gender							
Male	348	40.5%	135	36.2%	213	43.7%	0.030
Female	512	59.5%	238	63.8%	274	56.3%	

Family income							
<2 BMMS*	294	34.2%	120	32.2%	174	35.7%	0.130
2–4.9 BMMS*	286	33.2%	118	31.6%	168	34.5%	
≥5 BMMS*	200	23.3%	100	26.8%	100	20.5%	
Didn't know/no answer	80	9.3%	35	9.4%	45	9.3%	

Nutritional status							
Thinness	23	2.6%	8	2.2%	15	3.1%	0.544
Adequate	682	79.3%	304	81.5%	378	77.6%	
Overweight	126	14.7%	50	13.4%	76	15.6%	
Obesity	29	3.4%	11	2.9%	18	3.7%	

Fruit/vegetable intake							
Adequate	94	10.9%	43	11.5%	51	10.5%	0.660
Inadequate	766	89.1%	330	88.5%	436	89.5%	

Stages of change							
Precontemplation	376	43.7%	159	42.6%	217	44.6%	0.377
Contemplation	117	13.6%	46	12.3%	71	14.6%	
Preparation	273	31.7%	125	33.5%	148	30.4%	
Action	16	1.9%	8	2.1%	8	1.6%	
Maintenance	78	9.1%	35	9.4%	43	8.8%	

Total	**860**	**100%**	**373**	**100%**	**487**	**100%**	

*BMMS: Brazilian minimum monthly salary.

**Table 2:** Comparability between the intervention and control groups regarding fruit and vegetable intake and stages of change after the proposed intervention (analysis of the repeated measurements model using weighted minimum squares).

Variables	Control Group		Intervention Group		*P *value
	*n*	%	*n*	%	
Fruit/vegetable intake					
Adequate	27	8.4%	42	9.4%	0.626
Inadequate	296	91.6%	406	90.6%	

Stages of change					
Precontemplation	134	41.5%	195	43.5%	0.905
Contemplation	67	20.7%	89	19.9%	
Preparation	95	29.4%	122	27.2%	
Action	5	1.6%	9	2.0%	
Maintenance	22	6.8%	33	7.4%	

Total	**323**	**100%**	**448**	**100%**	

**Table 3:** Comparability between the intervention and control groups regarding decisional balance for benefits (“pros” scores) and barriers (“cons” scores) and self-efficacy, adjusted for sex and age, after the proposed intervention (analysis of covariance, ANCOVA).

Variables	Control Group		Intervention Group		*P *value
	Mean	Standard Error	Mean	Standard Error	
Pros scores	28.73	0.17	28.97	0.14	0.2885
Cons scores	13.50	0.16	13.33	0.13	0.4153
Self-efficacy scores	31.62	0.20	31.69	0.17	0.7749
